# Long-Term Mortality Analysis in Parkinson's Disease Treated with Deep Brain Stimulation

**DOI:** 10.1155/2014/717041

**Published:** 2014-03-03

**Authors:** Sofia Rocha, Ana Monteiro, Paulo Linhares, Clara Chamadoira, Margarida Ayres Basto, Carina Reis, Cláudia Sousa, Joana Lima, Maria José Rosas, João Massano, Rui Vaz

**Affiliations:** ^1^Department of Neurology, Hospital de Braga, Sete Fontes, São Victor, 4710-243 Braga, Portugal; ^2^Movement Disorders and Functional Surgery Unit, Centro Hospitalar São João, Alameda Prof. Hernâni Monteiro, 4200-319 Porto, Portugal; ^3^Department of Clinical Neurosciences and Mental Health, Faculty of Medicine, University of Porto, Alameda Prof. Hernâni Monteiro, 4200-319 Porto, Portugal; ^4^Department of Neurology, Hospital Pedro Hispano/ULS Matosinhos, Rua Dr. Eduardo Torres, Senhora da Hora, 4464-513 Matosinhos, Portugal

## Abstract

*Background.* Few data have been published regarding long-term mortality in patients with Parkinson's disease treated with DBS. *Methods.* This study analyzed long-term mortality rates, causes, and correlates in PD patients treated with DBS. *Results.* 184 consecutive patients were included; mean follow-up was 50 months. Fifteen deaths occurred (total 8.15%, annual mortality rate 1.94%). Mean age at disease onset and at surgery was 48 ± 2.4 and 63 ± 1.6 years, respectively. Mean disease duration until death was 21 ± 7.8 years. Most deaths related to stroke, myocardial infarction, other vascular/heart disorders, or severe infection; one suicide was recorded. Deceased PD patients were mostly male and had lower motor benefit after DBS, but univariate analysis failed to show significant differences regarding gender and motor benefit. Survival was 99% and 94% at 3 and 5 years. *Conclusions.* Long-term survival is to be expected in PD patients treated with DBS, possibly higher than previously expected. Death usually supervenes due to vascular events or infection.

## 1. Introduction

Parkinson's disease is a common, disabling neurodegenerative disorder. Motor symptoms and quality of life improve significantly under adequate treatment, although motor complications commonly arise, typically a few years after the onset of dopaminergic therapy [1]. Several studies have looked into mortality in PD and found a higher risk of death as compared to the general population, even after the introduction of levodopa [2, 3]. Deep brain stimulation (DBS) is an effective procedure in Parkinson's disease (PD) with motor complications [4]. It is safe and surgery-related mortality is low [5]. However, little is known about the long-term mortality in this population, in particular regarding death rates and causes, as few data have been published [6–8]. The recent publication of the EARLYSTIM trial results emphasizes the importance of analyzing this issue in depth, as the number of candidates for DBS will predictably increase in the near future [9]. The aims of this research paper were to evaluate the rates and causes of death in PD patients treated with DBS, up to ten years after surgery.

## 2. Patients and Methods

Patients enrolled in this study had been consecutively submitted to DBS at the Movement Disorders and Functional Surgery Unit of a large University Hospital between October 2002 and November 2012. All patients were examined before and one month after surgery and then every 6 months. Unified Parkinson's Disease Rating Scale (UPDRS) was assessed before surgery with and without L-dopa medication. After the procedure UPDRS was determined, with stimulation turned on and off. Behavior, mood, activities of daily living, disability, and complications from therapy were assessed using UPDRS (parts I, II, and IV), Schwab and England scale, Beck Depression Inventory, and Geriatric Depression Scale. All patients underwent a comprehensive neuropsychological evaluation prior to surgery and postoperatively at 6 months, 18 months, and 5 years. Neuropsychological test batteries were administered by a trained neuropsychologist. Instruments included the Mini-Mental State Examination, the Frontal Assessment Battery, the Clock Drawing Test, the Dementia Rating Scale, verbal fluency (semantic and phonemic), digit span, associative verbal memory, and visual memory tasks from the Wechsler Memory Scale, the Stroop Test, the Trail Making Test, and the Wisconsin Card Sorting Test. All cognitive assessments before and after surgery were performed while the patients were at a state of pragmatically defined “on.” Postoperative assessments were performed with the stimulators turned on. Adverse events following surgery (including date and cause of death) were recorded during the inpatient period and at each outpatient visit. In addition, general demographic (e.g., age, gender) and clinical data (e.g., disease duration, phenotype, medication type, and dosage) were documented for each patient.

Widely accepted standard inclusion and exclusion criteria for DBS have been observed [10, 11], All subjects met the following criteria: (i) Parkinson's disease diagnosed according to the United Kingdom Parkinson's Disease Society Brain Bank criteria (in our patients the diagnosis had been established for at least 5 years); (ii) at least 50% improvement in motor symptoms after an acute levodopa challenge following complete medication withdrawal for at least 12 hours; (iii) troublesome motor fluctuations and dyskinesias, despite optimal medical therapy adjusted by expert in PD; (iv) clinical examination fully consistent with PD; (v) age up to 70 years, with some degree of flexibility; (vi) absence of dementia or major uncontrolled psychiatric disorders, including suicidal ideation; (vii) absence of neurosurgical, neuroradiological, or general medical contraindications.

Data used in this study concerns follow-up until death or November 2012. No patient was lost to follow-up. Causes of death were determined from confirmed clinical data, except in one patient in whom autopsy was carried out.

Kaplan-Meier survival curves were constructed using death (any cause) as the endpoint. Beyond a descriptive analysis, we performed a comparative analysis using Cox regression to assess differences by gender, disease type, age at motor symptom onset, age at the time of surgery, disease duration until surgery, UPDRS before surgery with and without L-dopa medication, and motor benefit one month after surgery. A descriptive, comparative, and survival analysis was performed, using STATA and SPSS (version 20.0).

## 3. Results

Between October 2002 and November 2012, 184 PD patients were treated with bilateral deep brain stimulation (181 subthalamic nuclei, 3 globus pallidus internus). Fifteen deaths occurred during the follow-up period (mean 50 months, global mortality 8.15%, and annual mortality rate 1.94%), none of which occurring within the first month after surgery.

### 3.1. Characteristics of the Whole PD Population Treated with DBS

Sixty-one percent of PD patients treated with DBS were men (*n* = 113). Mean age at disease onset was 47 ± 9 years (18–64) and mean age at surgery was 60 ± 8 years (33–73). Mean disease duration until surgery was 14 years (5–48). Sixty percent of patients (*n* = 111) presented akinesia and rigidity as the main disease motor symptoms. Presurgical UPDRS part III average score was 46 ± 11 off medication and 15 ± 7 at the best on state. The average motor benefit in the first month after surgery, based on UPDRS part III before surgery without medication and UPDRS part III after surgery on stimulation and off medication, was 74 ± 14%. There were no significant differences between STN and GPi stimulated patients regarding demographic data, UPDRS scores, or levodopa responsiveness.

### 3.2. Characteristics of Deceased Patients

Mean age at disease onset and at the time of surgery was 48 ± 2.4 (28–59) and 63 ± 1.6 years (51–71), respectively. Mean disease duration until surgery and until death was 15 ± 1.6 (7–35) and 21 ± 7.8 (11–35) years, respectively. All deceased patients had received subthalamic DBS. Average time elapsed between surgery and death was 65 ± 33.1 months (min 2; max 115), and mean age at the time of death was 67 ± 6.3 (54–76) years. Preoperative UPDRS-III average score was 46 (33–62) off medication and 12 (7–20) at the best on state. The mean motor benefit one month after surgery was 62% (46–85).

**Figure 1:**
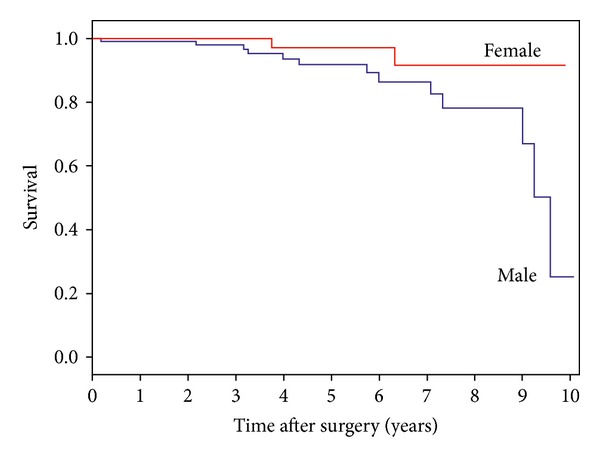
Survival of PD patients by gender. Cox regression indicates no statistical difference between groups.

**Figure 2:**
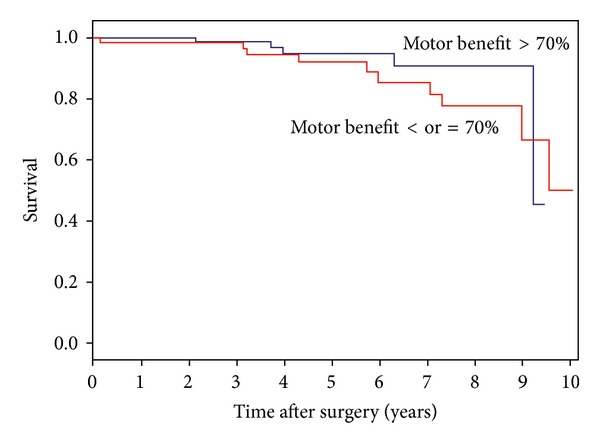
Survival of PD patients by motor benefit following DBS. Cox regression indicates no statistical difference between groups.

Eight of the deceased patients (53%) had clinical criteria for dementia [12], at least at the last observation.  Table 1 shows the comparison between deceased and alive patients, regarding baseline features. Deaths were mostly attributed to vascular events (*n* = 6) and respiratory infections (*n* = 5); one suicide occurred 39 months after surgery (Table 2). Deceased PD patients were mostly men (*n* = 13 or 87% of total deaths, *P* = 0.032) and had gained less motor benefit from surgery (62% versus 76%, *P* = 0.0004). However, univariate analysis (Cox regression and Kaplan-Meier survival analysis) did not show significant differences regarding gender (HR = 3.740, *P* = 0.083; IC 95% (0.840–16.654)) or motor benefit (cut-off at 70%: HR = 1.685, *P* = 0.362; IC 95% (0.549–5.174)) between deceased and nondeceased groups (Figures 1 and 2).

**Figure 3:**
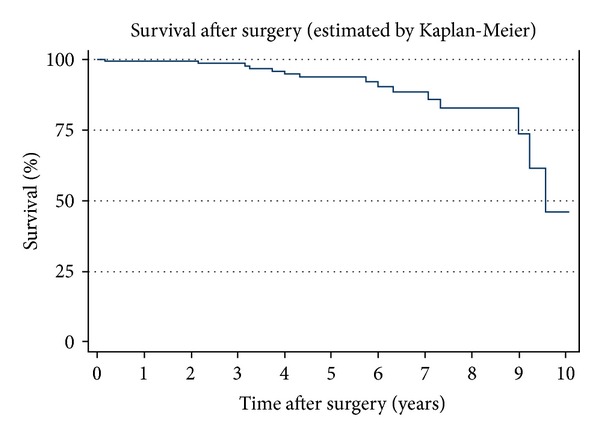
Kaplan-Meier survival curve for the studied population.

Age at surgery higher than 65 years correlated with higher mortality risk (HR = 3.053, *P* = 0.040; IC a 95% (1.53–8.849)). Survival in this series was 99% at 3 years, 94% at 5 years, and 88% at 7 years after surgery (Figure 3).

## 4. Discussion

To the best of our knowledge this is the largest series published so far analyzing long-term mortality in DBS-treated PD patients [4, 6–8, 13].

Survival in this series seems somewhat higher than previously reported (Toft et al. found survival rates of 97% at 3 years and 90% at 5 years; Schüpbach et al. reported 97% at 2 years and 89% at 5 years), although it is not possible to calculate whether the difference is significant [6–8]. Anyway, the annual mortality rate reported in this series is clearly lower than that described by Wider et al. (8.5%) [8].

Mean age at death (67 years) was higher than that reported by Schüpbach et al. (63 years), although our patients had a higher mean age at surgery (60 versus 57 years) and a slightly higher mean disease duration until surgery (14 versus 13 years) [7]. The mean follow-up time between surgery and death (65 months) was also higher than previously described (Wider et al. 42 months, Schüpbach et al. 45, and Toft et al. 42) [6–8]. Taken altogether, our results suggest that survival following DBS in PD might be higher than previously estimated.

In this series, age at surgery above 65 years seems to be a predictive mortality factor, as reported by other authors [6, 8]. However, we find ourselves unable to relate this finding to the procedure, and this could be due to natural history only (i.e., older patients are at higher risk of death per se). Preoperative UPDRS-III on and off medication was not different between deceased and nondeceased patients, in contrast to the results reported by Toft et al., who found a relationship between mortality and higher UPDRS score off medication preoperatively [6]. Age at disease onset, disease duration until surgery, and the phenotype of the disease were also not different between groups. Our PD deceased patients were mostly men, which parallels with previously published data reporting greater mortality in male PD patients who did not have DBS [14]. Moreover, deceased patients had lower motor benefit one month after surgery (62% versus 76%, *P* = 0.0004). One might speculate that axial symptoms in these patients could be more severe thus predisposing to falls, but only one death (out of 15) was related to falling. Otherwise, there were no statistically significant differences between deceased and nondeceased groups with regard to gender or motor benefit. Also, it is not possible to accurately compare STN and GPi groups in this series, due to the very small number of patients in the latter (181 versus 3).

In this series infection was a common cause of death, mainly of respiratory origin, which also stands in agreement with published studies describing pneumonia as an important cause of death in PD [2, 14]. In our series there were no deaths within the first month after surgery, nor any deaths related to the procedure or hardware complications, which stands in agreement with known DBS safety data. There was one suicide (0.54% of all DBS cases), which stands in accordance with the results from the largest multicenter study published so far on this topic in DBS-treated PD patients [15].

Of note, most deceased patients in this study fulfilled criteria for dementia, which has been associated with a higher mortality risk in PD [16].

Our study is limited by the absence of a control group of clinically similar PD patients who did not have DBS (e.g., due to patient refusal to have surgery), in order to compare survival and death causes. Nonetheless, we feel intrigued by the fact that long-term survival seems relatively high for a population of advanced PD patients with long disease duration and wonder whether DBS has any positive effect on survival in PD.

In summary, we found that long-term mortality rates might be lower than previously estimated, and high survival rates are to be expected following DBS in PD, despite the fact that patients already present motor complications and long disease duration at the time of surgery. Mortality is higher among PD male patients or those gaining less motor benefit from surgery, although neither gender nor motor benefit seems to predict death. Older age at surgery correlates with mortality, but this cannot be associated with the procedure itself, and could be simply related to the fact that older people are at higher risk of death. Death in these patients is largely unrelated to PD or surgical complications, as vascular events and pneumonia lead the causes, which stands in line with published epidemiological data in PD. Future research studies should prospectively assess the effect of DBS on patient survival in PD, as compared to best medical therapy.

## Figures and Tables

**Table 1:** Comparison between deceased patients and survivors.

	Deceased patients	Nondeceased patients	*P* value
Male gender: *n* (%)	13 (86.67)	100 (59.17)	**0.032**
Age at disease onset (mean, years)	48.07 ± 2.41	46.74 ± 0.69	0.707
Akinetic-rigid phenotype: *n* (%)	8 (53.33)	103 (60.95)	0.564
Age at the time of DBS (mean, years)	62.8 ± 1.62	60.23 ± 0.59	0.894
Disease duration until DBS (mean, years)	14.53 ± 1.59	13.42 ± 0.46	0.751
UPDRS part III before surgery without L-dopa medication (mean)	45.6 ± 2.09	46.34 ± 0.84	0.399
UPDRS part III before surgery with L-dopa medication (mean)	12.47 ± 1.20	14.90 ± 0.51	0.083
Motor benefit after surgery (mean, %)	62.4 ± 5.26	75.52 ± 0.96	**0.0004**

**Table 2:** Deceased PD patients treated with DBS. M: male, F: female.

Patient	Gender	Time between surgery and death (months)	Cause of death	Comorbidities
1	M	48	Metastasized colon cancer	Hypertension Ischemic stroke
2	F	45	Myocardial infarction	Urinary lithiasis
3	M	52	Pneumonia, sepsis	Hypertension Urinary lithiasis Dementia
4	M	69	Peritonitis, sepsis	Kidney cancer Dementia
5	M	39	Suicide	—
6	M	38	Hemorrhagic stroke	Dyslipidemia Dementia
7	M	108	Hemorrhagic stroke	Prostatic cancer
8	F	76	Traumatic brain injury	Depression Dementia
9	M	72	Ischemic stroke	Diabetes mellitus Hypertension Dementia
10	M	115	Pneumonia	—
11	M	26	Myocardial infarction	Herniated cervical disk
12	M	88	Pneumonia	Diabetes mellitus Prostatic benign hyperplasia Dementia
13	M	111	Pneumonia	Ischemic stroke Dementia
14	M	2	Cardiomyopathy	—
15	M	85	Mesenteric thrombosis	Traumatic subdural hemorrhage Dementia
